# Supplementary material for: Balancing reaction-diffusion network for cell polarization pattern with stability and asymmetry
Source: eLife. 2025 Jul 22;13:RP96421. doi: 10.7554/eLife.96421 (PMC12283076; doi:10.7554/eLife.96421)
Supplement: Source code 1. [file elife-96421-code1.zip › PolarSim/GuideBook.docx]

**Instructions for *PolarSim***

**2.1. Introduction**

*PolarSim* is a graphical user interface (GUI) on *Matlab* 2022b [The MathWorks Inc., 2022b] for simulating the evolution of cell polarization patterns. Based on the reaction-diffusion model, the GUI allows users to compute the behaviors of cell polarization networks in different biological scenarios. All the simulations are tested with a 12th Gen Intel(R) Core(TM) i7-1260P CPU.

**2.2. Tutorials**

·Download the folder “PolarSim” from https://github.com/YixuanChen0726/Cell-Polarization/tree/main/PolarSim.

- Open *Matlab* under the “PolarSim” folder path and execute script “GUI.m”. Click “Run” and then an interactive interface pops up (Appendix 2 – figure 1).


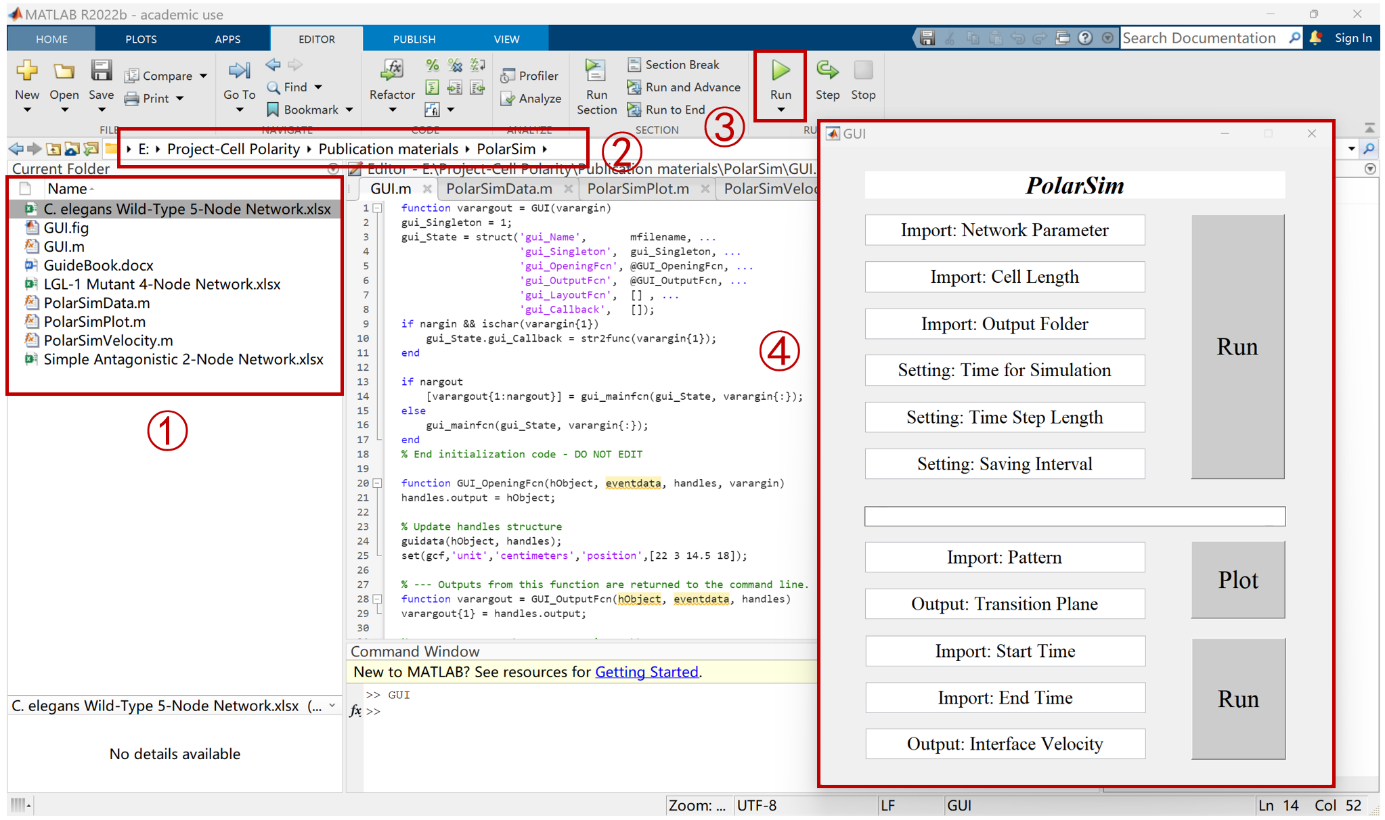


**Appendix 2** – **figure 1.** The instructions to open the *PolarSim* GUI. ① The files in the folder “PolarSim”. ② Open the *Matlab* under the path of the folder “PolarSim” and double-click to open “GUI.m”. ③ Click “Run” to open the *PolarSim* GUI shown by ④.

- With the following parameters inputted, the GUI gives out a group of “Pattern_*.mat” files containing pattern information.

(1) Import: Network Parameter.

We give out three examples “Simple Antagonistic 2_Node Network.xlsx”, “LGL-1 Mutant 4-Node Network.xlsx” and “C. elegans Wild-Type 5-Node Network.xlsx” in the folder “PolarSim”, respectively representing the typical networks in this paper (Appendix 2 – figure 2). The Excel table for parameter value assignments should follow the format below:

|  | Node A | Node B | … | Node N |
| --- | --- | --- | --- | --- |
|  | Location (a or p)/  $[A_{c}]$/$\gamma_{A}$/$\alpha_{A}$ | Location (a or p)/  $[B_{c}]$/$\gamma_{B}$/$\alpha_{B}$ | … | Location (a or p)/  $[N_{c}]$/$\gamma_{N}$/$\alpha_{N}$ |
| Node A | $q_{A1}^{A}$/$q_{A2}^{A}$/$n_{\mathrm{Aq}}^{A}$/$k_{A1}^{A}$/$k_{A2}^{A}$/$n_{\mathrm{Ak}}^{A}$ | $q_{B1}^{A}$/$q_{B2}^{A}$/$n_{\mathrm{Bq}}^{A}$/$k_{B1}^{A}$/$k_{B2}^{A}$/$n_{\mathrm{Bk}}^{A}$ | … | $q_{N1}^{A}$/$q_{N2}^{A}$/$n_{\mathrm{Nq}}^{A}$/$k_{N1}^{A}$/$k_{N2}^{A}$/$n_{\mathrm{Nk}}^{A}$ |
| Node B | $q_{A1}^{B}$/$q_{A2}^{B}$/$n_{\mathrm{Aq}}^{B}$/$k_{A1}^{B}$/$k_{A2}^{B}$/$n_{Ak}^{B}$ | $q_{B1}^{B}$/$q_{B2}^{B}$/$n_{\mathrm{Bq}}^{B}$/$k_{B1}^{B}$/$k_{B2}^{B}$/$n_{\mathrm{Bk}}^{B}$ | … | $q_{N1}^{B}$/$q_{N2}^{B}$/$n_{\mathrm{Nq}}^{B}$/$k_{N1}^{B}$/$k_{N2}^{B}$/$n_{\mathrm{Nk}}^{B}$ |
| … | … | … | … | … |
| Node N | $q_{A1}^{N}$/$q_{A2}^{N}$/$n_{\mathrm{Aq}}^{N}$/$k_{A1}^{N}$/$k_{A2}^{N}$/$n_{\mathrm{Ak}}^{N}$ | $q_{B1}^{N}$/$q_{B2}^{N}$/$n_{\mathrm{Bq}}^{N}$/$k_{B1}^{N}$/$k_{B2}^{N}$/$n_{\mathrm{Bk}}^{N}$ | … | $q_{N1}^{N}$/$q_{N2}^{N}$/$n_{\mathrm{Nq}}^{N}$/$k_{N1}^{N}$/$k_{N2}^{N}$/$n_{\mathrm{Nk}}^{N}$ |

**Appendix 2 - table 1.** The instructions for the format of Network Parameter in an Excel table. In the 1^st^ row and 1^st^ column, “Node N” represents the name of the node. The 2^nd^ row explains the characteristic parameters of each node (*i.e.* location, cytoplasmic concentration, basal on-rate, and basal off-rate as listed from left to right). The interaction parameters start from the 3^rd^ row and 2^nd^ column, where the *i*^th^ row and *j*^th^ column describe the activation/inhibition effect from Node *j* to Node *i*. Note that $q_{Y2}^{X}$ and $k_{Y2}^{X}$ should be set to 0 when no activation or inhibition is exerted on X from Y respectively. “Location” should be assigned with the string “a” or “p” while the description of the other parameters is detailed in Appendix 1 – table 1.


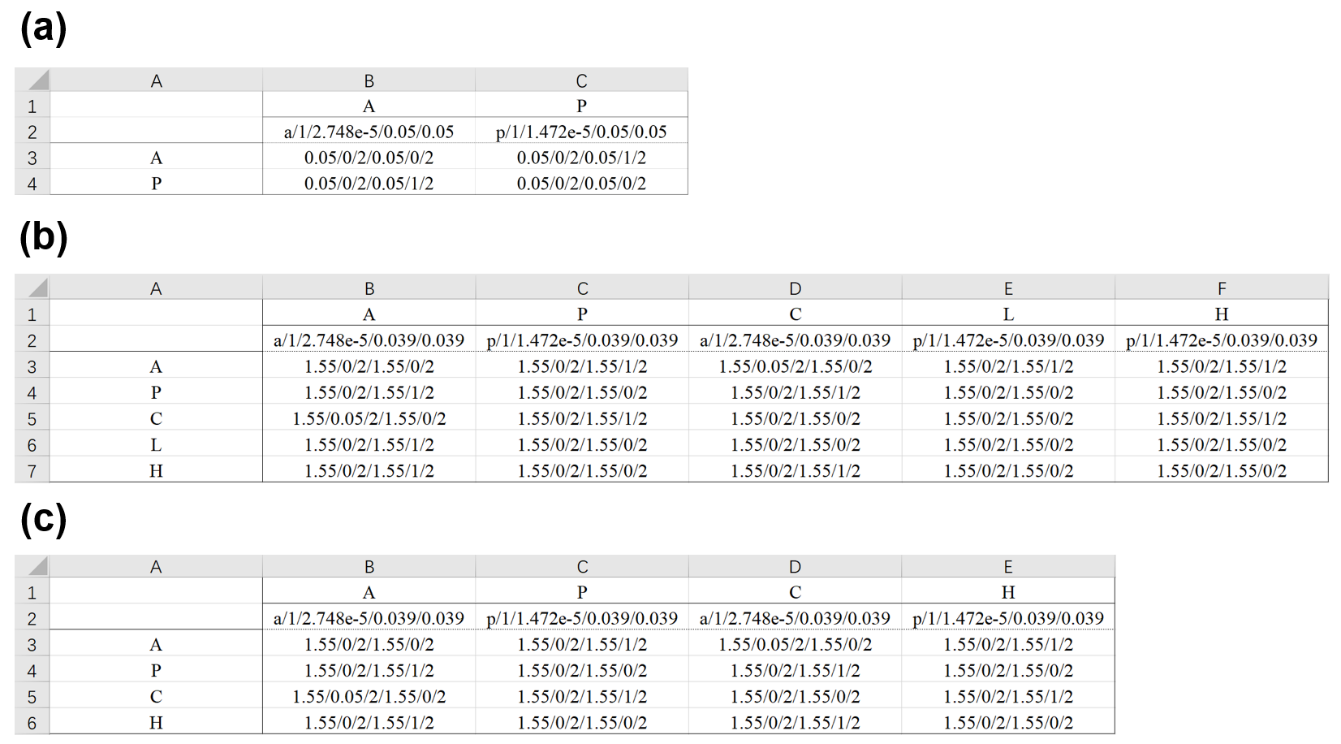


**Appendix 2** – **figure 2.** The examples of network parameters. (a) “Simple Antagonistic 2-Node Network.xlsx” lists the parameters in Figure 1a. (b) “C. elegans Wild-Type 5-Node Network.xlsx” lists the parameters for the benchmark point $P^{*}\left( \gamma^{*}=0.039, k_{1}^{*}=1.55, q_{2}^{*}=0.05 \right)$ in Figure 6b. (c) “LGL-1 Mutant 4-Node Network.xlsx” lists the parameters as in (b) but with the Node $[L]$ knocked out.

(2) Import: Cell Length

We take “0.5” as an example. Any positive number is allowed in this box. The effects of cell length on cell polarization patterns are shown in Appendix 2 – figure 6.

(3) Import: Output Folder

Give a folder name for storing the output results (*e.g.* “Output 2-Node”)

(4) Setting: Time for Simulation

Simulation duration “500” is used in this paper. Any positive number is allowed in this box.

(5) Setting: Time Step Length

We take “1” as an example. Larger values are not recommended, in consideration of possible error accumulation, and one may try a smaller step length while the error tolerance is exceeded.

(6) Setting: Saving Interval

It must be an integer multiple of the time step length. The time point will be saved in this designated interval and can be used for pattern plotting later.

- Click “Run” in the interface, and then its status on the progress bar is shown (Appendix 2 – figure 3a). A folder named “Import: Output Folder” is generated in the current path to store the output “Pattern_*.mat” data containing the molecular species or node’s name, location, and concentration distribution on the cell membrane, where “*” denotes the *in silico* time corresponding to each file (Appendix 2 – figure 4a – right).
- Import the pathway of the file outputted by *PolarSim* into the box “Import: Pattern”.
- Click “Plot” and then a figure pops up to show the cell polarization pattern while the position of transition plane appears in the box “Output: Transition Plane”.
- Import the pathway of two files outputted by *PolarSim* into the box “Import: Start Time” and “Import: End Time”.
- Click “Run” and then the mean interface velocity between two input time points appears in the box “Output: Interface Velocity”.


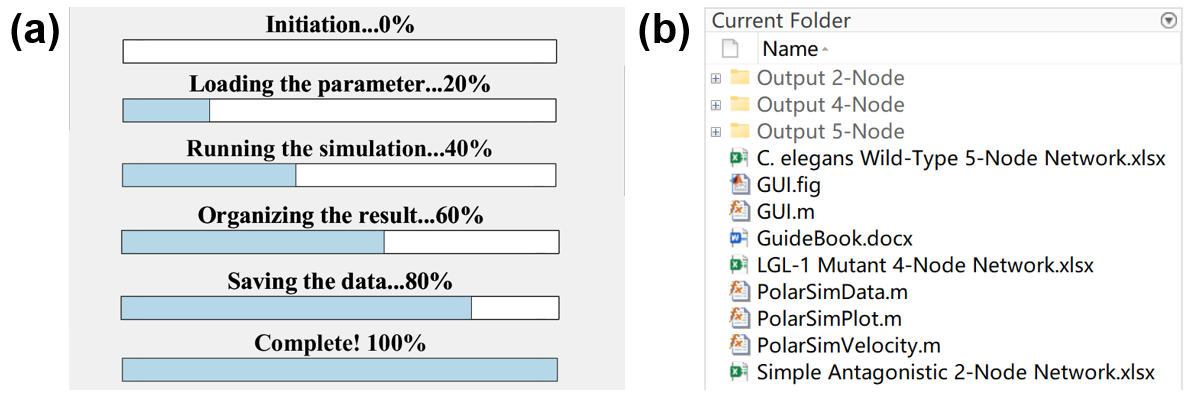


**Appendix 2 – figure 3.** (a) The progress bar showing the running progress. (b) The output subfolders in the folder “PolarSim”.


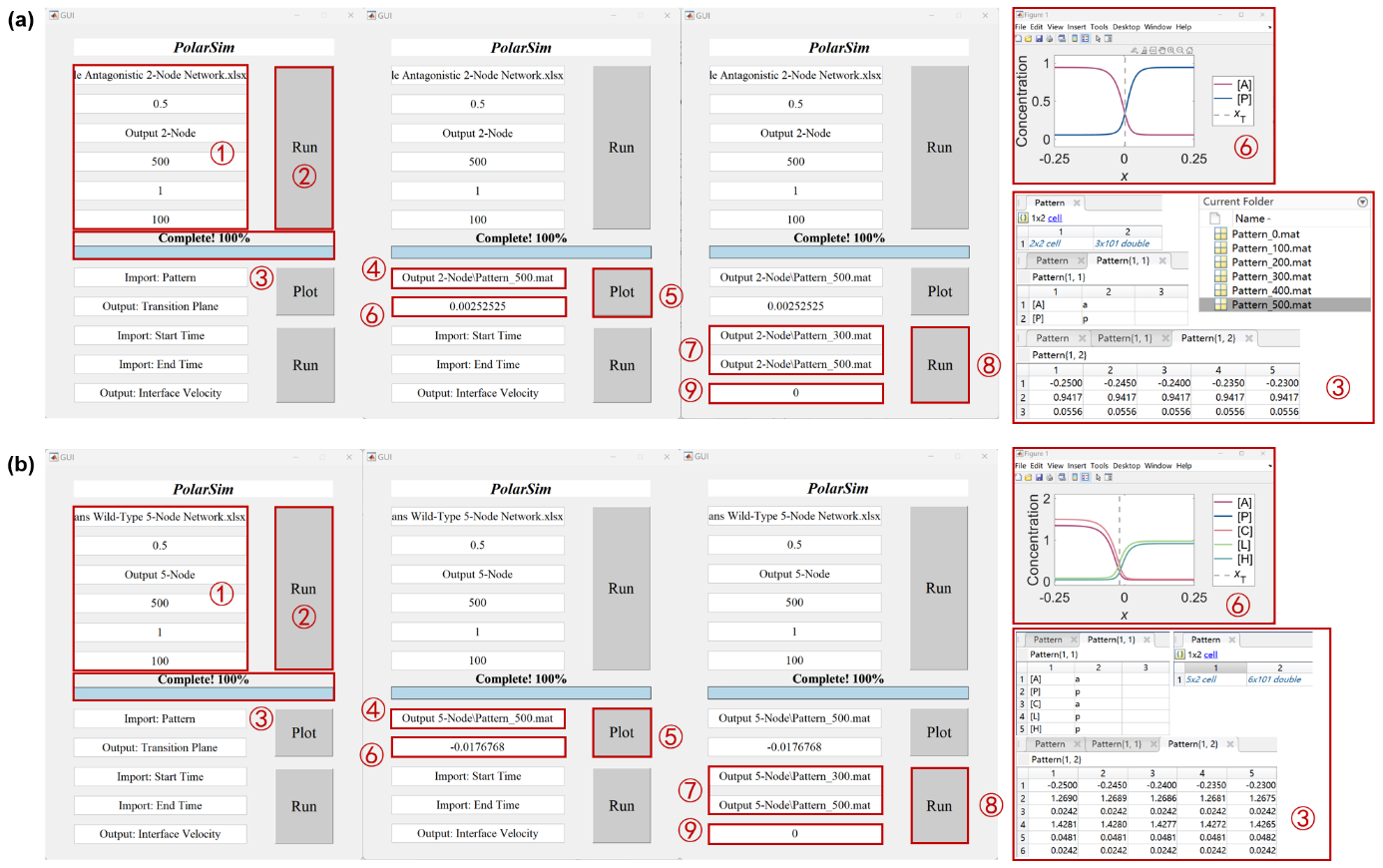


**Appendix 2 – figure 4.** The results of Example 1 (“Simple Antagonistic 2-Node Network.xlsx”) and Example 2 (“C. elegans Wild-Type 5-Node Network.xlsx”) of *PolarSim*. (a) The flow chart for computing Example 1: ① input parameters; ② click “Run”; ③ the simulation is completed with a progress bar shown and files saved in the folder “Output 2-Node”; the file “Pattern_500.mat” is used to show the data format on the right, where the first part stores the molecular species or nodes’ names and locations while the second part stores their spatial concentration distributions on the cell membrane; ④ input the pathway of the outputted pattern file; ⑤ click “Plot”; ⑥ the simulation is completed with a figure shown and the position of transition plane given in the box “Output: Transition Plane”; ⑦ input the pathway of the two outputted files “Pattern_*.mat” at different time points into “Import: Start Time” and “Import: End Time”, where “*” denotes the start time and end time respectively; ⑧ click “Run”; ⑨ the simulation is completed with the interface velocity given in the box “Output: Interface Velocity”. (b) The same as (a) but for the “C. elegans Wild-Type 5-Node Network.xlsx”.

**2.3. Examples**

· **Example 1**: “Simple Antagonistic 2-Node Network.xlsx” simulation as Figure 1a.

(1) Input the following parameters: “Simple Antagonistic 2-Node Network.xlsx” into “Import: Network Parameter”; “0.5” into “Import: Cell Length”; “Output 2-Node” into “Import: Output Folder”; “500” into “Setting: Time for Simulation”; “1” into “Setting: Time Step Length” and “100” into “Setting: Saving Interval”. (Appendix 2 – figure 4a – ①)

(2) Click “Run”, and then six “Pattern_*.mat” files at different time points are saved in the subfolder “Output 2-Node” (Appendix 2 – figure 4a – ②-③).

(3) Input “Output 2-Node\Pattern_500.mat” into the box “Import: Pattern”, and a figure at $t=500$ appears. Then the position of transition plane $x_{T}=0.00252525$ is given in the box “Output: Transition Plane” (Appendix 2 – figure 4a – ④-⑥).

(4) Input “Output 2-Node\Pattern_300.mat” and “Output 2-Node\Pattern_500.mat” into the box “Import: Start Time” and “Import: End Time” respectively. Then the interface velocity between $t=300$ and $t=500$ is given in the box “Output: Interface Velocity” (Appendix 2 – figure 4a – ⑦-⑨). The 2-node network approaches a stable polarized pattern with its interface velocity being 0.

· **Example 2**: “C. elegans Wild-Type 5-Node Network.xlsx” simulation as Figure 6b.

(1) Input the following parameters: “C. elegans Wild-Type 5-Node Network.xlsx” into “Import: Network Parameter”; “0.5” into “Import: Cell Length”; “Output 5-Node” into “Import: Output Folder”; “500” into “Setting: Time for Simulation”; “1” into “Setting: Time Step Length” and “100” into “Setting: Saving Interval” (Appendix 2 – figure 4b – ①).

(2) Click “Run”, and then six “Pattern_*.mat” files at different time points are saved in the subfolder “Output 5-Node” (Appendix 2 – figure 4b – ②-③).

(3) Input “Output 5-Node\Pattern_500.mat” into the box “Import: Pattern”, and a figure at $t=500$ appears. Then the position of transition plane $x_{T}=-0.0176768$ is given in the box “Output: Transition Plane” (Appendix 2 – figure 4b – ④-⑥).

(4) Input “Output 5-Node\Pattern_300.mat” and “Output 5-Node\Pattern_500.mat” in the box “Import: Start Time” and “Import: End Time” respectively. Then the interface velocity between $t=300$ and $t=500$ is given in the box “Output: Interface Velocity” (Appendix 2 - figure 4b – ⑦-⑨). The 5-node network approaches a stable polarized pattern with its interface velocity being 0.

· **Example 3**: “LGL-1 Mutant 4-Node Network.xlsx” simulation, originated from “C. elegans Wild-Type 5-Node Network.xlsx” but with the Node $[L]$ knocked out.

(1) Input the following parameters: “LGL-1 Mutant 4-Node Network.xlsx” into “Import: Network Parameter”; “0.5” into “Import: Cell Length”; “Output 4-Node” into “Import: Output Folder”; “1000” into “Setting: Time for Simulation”; “1” into “Setting: Time Step Length” and “100” in “Setting: Saving Interval” (Appendix 2 – figure 5a).

(2) Click “Run”, and then 11 “Pattern_*.mat” files at different time points are saved in the subfolder “Output 4-Node”.

(3) Input “Output 4-Node\Pattern_500.mat” into the box “Import: Pattern”, and then a figure of LGL-1 Mutant 4-Node Network at $t=500$ appears with the transition plane close to the posterior pole. Then the position of transition plane $x_{T}=0.207071$ is given in the box “Output: Transition Plane” (Appendix 2 – figure 5a).

(4) Input “Output 4-Node\Pattern_300.mat” and “Output 4-Node\Pattern_500.mat” in the box “Import: Start Time” and “Import: End Time” respectively. Then the interface velocity between $t=300$ and $t=500$ is given in the box “Output: Interface Velocity” (Appendix 2 – figure 5a). The interface of the 4-node network keeps moving toward the posterior with $v_{I}=0.000429293$.

(3) Input “Output 4-Node\Pattern_1000.mat” in the box “Import: Pattern”, and then a figure at $t=1000$ appears. The string “Transition plane doesn’t exist” appears in the box “Output: Transition Plane” as the pattern reaches a homogeneous state dominated by $[A]$ and $[C]$ (Appendix 2 – figure 5b).

(4) Input “Output 4-Node\Pattern_800.mat” and “Output 4-Node\Pattern_1000.mat” in the box “Import: Start Time” and “Import: End Time” respectively. Then the interface velocity between $t=800$ and $t=1000$ doesn’t exist with the string “It isn’t a polarized pattern” appearing in the box “Output: Interface Velocity” to hint (Appendix 2 – figure 5b). Note that the interface velocity can’t be calculated when either pattern at start time or end time is homogeneous.


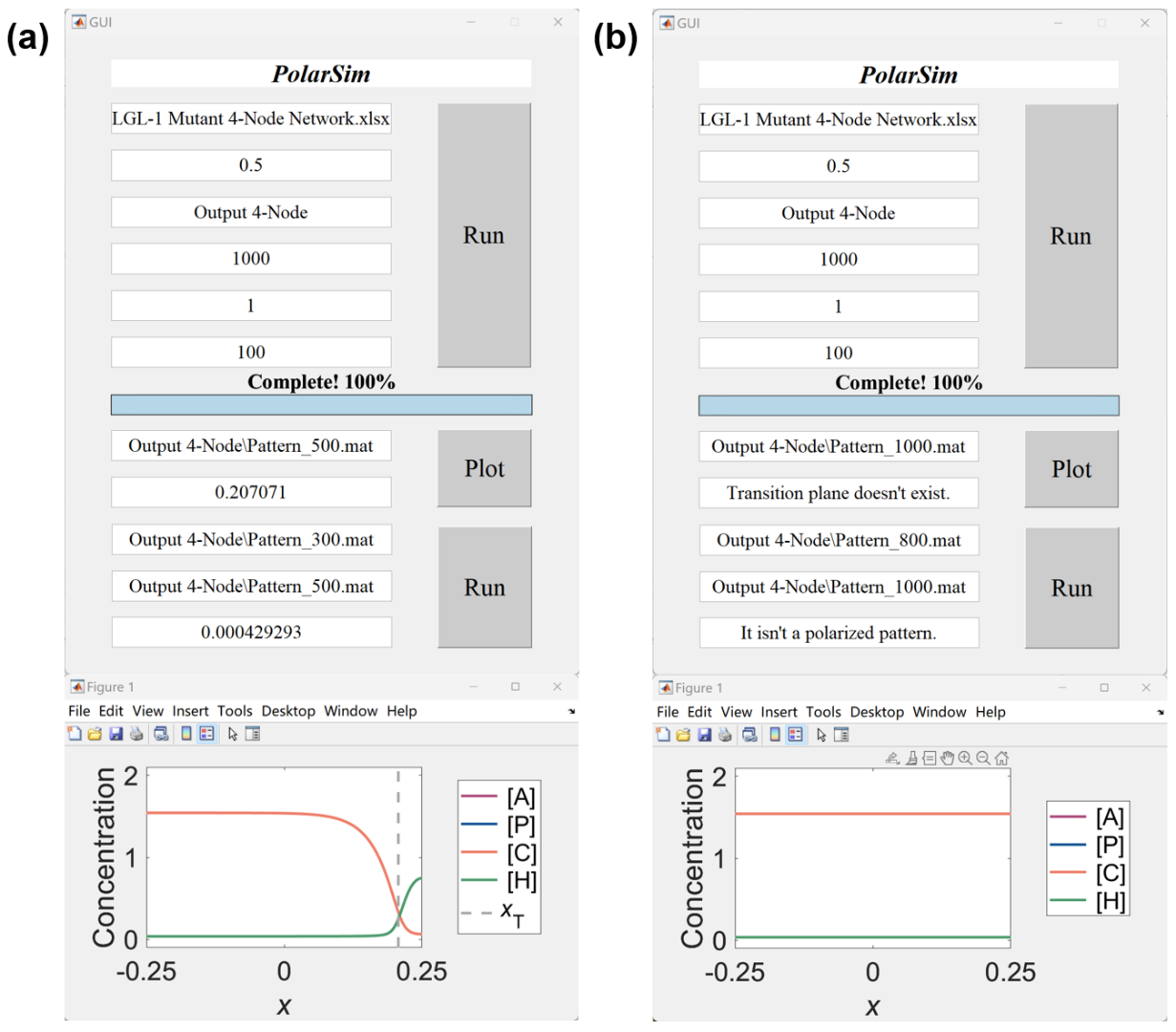


**Appendix 2 – figure 5.** The results of Example 3 (“LGL-1 Mutant 4-Node Network.xlsx”) of *PolarSim*. (a) The transition plane is shown at $t=500$, with the value $x_{T}=0.207071$. The interface velocity is calculated between $t=300$ and $t=500$, with the value $v_{I}=0.000429293$ representing an unstable pattern (top). The figure is plotted at $t=500$ (bottom). (b) The transition plane and the figure are shown at $t=1000$and the interface velocity is calculated between $t=800$ and $t=1000$. The pattern collapses to a homogeneous state with $[A]$ and $[C]$ invading the posterior domain at $t=1000$, and thereby the transition plane doesn’t exist and the interface can’t be calculated.

**2.4. Extensive Application**

Our *PolarSim* is extensively applicable to similar biological systems. Here, we take the cell size (length) as an exemplary research target to study how the concentration distribution on the cell membrane depends on it. Different cell lengths are applied to the Example 1 (“Simple Antagonistic 2-Node Network.xlsx”) and Example 2 (“C. elegans Wild-Type 5-Node Network.xlsx”) to see whether there is a cell size threshold limiting cell polarization as discovered before [Hubatsch et al., *Nat. Phys.*, 2019]. Patterns at $t=500$ are plotted with the cell length ranging from 0.1 to 0.5 in steps 0.1 (Appendix 2 – figure 6).


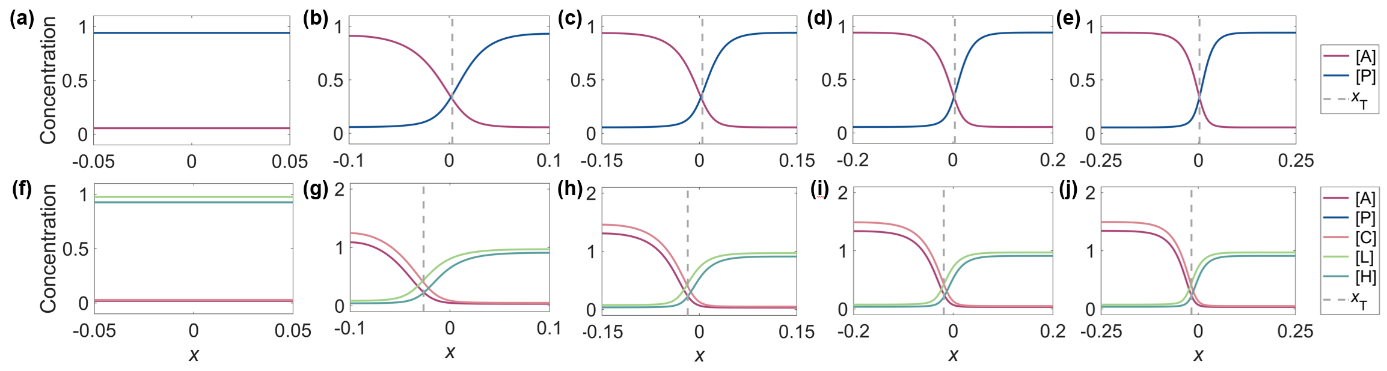


**Appendix 2 – figure 6.** The effects of cell size (length) on the cell polarization pattern. (a-e) The pattern of “Simple Antagonistic 2-Node Network.xlsx” at $t=500$. From left to right, the cell lengths are 0.1, 0.2, 0.3, 0.4 and 0.5, respectively. (f-j) The same as (a-e) but for the “C. elegans Wild-Type 5-Node Network.xlsx”.

The *PolarSim*-based simulations above indicate that the proper cell polarization demands a reasonable spatial scale, which to some extent gives an explicit constraint for the volume of a cell in reality when it needs to divide asymmetrically by reading out its cell polarization pattern interface [Hubatsch et al., *Nat. Phys.*, 2019]. It’s worth noting that our prediction of cell polarization pattern collapse (with less and less distinguishable interface) over cell size decrease aligns with previous experimental and theoretical research [Hubatsch et al., *Nat. Phys.*, 2019]. In all, *PolarSim* provides a user-friendly tool for more applications on the studies in cell polarization.

**2.5. Contact**

All the scripts of the *PolarSim* GUI have been uploaded onto GitHub https://github.com/YixuanChen0726/Cell-Polarization/tree/main/PolarSim. If there is any question, please contact Yixuan Chen (yixuanchen@stu.pku.edu.cn) or Guoye Guan ([guanguoye@gmail.com](mailto:guanguoye@gmail.com)) anytime.
